# What women say about their dysmenorrhea: a qualitative thematic analysis

**DOI:** 10.1186/s12905-018-0538-8

**Published:** 2018-03-02

**Authors:** Chen X. Chen, Claire B. Draucker, Janet S. Carpenter

**Affiliations:** Indiana University School of Nursing, 600 Barnhill Drive, NU E415, Indianapolis, IN 46202 USA

**Keywords:** Dysmenorrhea, Qualitative research, Women’s health, Pain, Patient preference, Menstruation

## Abstract

**Background:**

Dysmenorrhea is highly prevalent and is the leading cause of absence from school and work among women of reproductive age. Evidence suggests that dysmenorrhea may also be a risk factor for other chronic pain conditions. Limited research has examined women’s experience with dysmenorrhea using qualitative data. Research is warranted to address issues and needs that are important from women’s own perspectives. Therefore, the purpose of this study was to describe women’s salient thoughts about their experiences of dysmenorrhea.

**Methods:**

We analyzed data collected from an open-ended question within a cross-sectional survey study conducted in the United States. Using qualitative thematic analysis, free text responses to a question asking women to share their experience with dysmenorrhea were analyzed.

**Results:**

The sample consisted of 225 women who provided valid responses to the open-ended question. Six themes were identified: (1) The dysmenorrhea symptom experience varied among women; (2) The dysmenorrhea symptom experience varied across time, (3) A variety of factors influenced the dysmenorrhea symptom experience, (4) Dysmenorrhea symptoms could have a negative impact on the women’s daily lives, (5) Dysmenorrhea was not seen as a legitimate health issue by the women, health care providers, or society, and (6) Treatment for women with dysmenorrhea varied in acceptability and effectiveness.

**Conclusions:**

The findings of this study have important implications for dysmenorrhea symptom assessment and the development of personalized interventions to support dysmenorrhea management.

## Background

Dysmenorrhea is characterized by abdominal pain occurring just before and/or during menstruation [1]. Its prevalence among women of reproductive age ranges from 16 to 91% [2]. Dysmenorrhea is classified as primary if it occurs in the absence of underlying pathological findings or secondary if it is related to other conditions such as endometriosis, fibroids, or pelvic inflammatory diseases [1, 3, 4]. Without a pelvic examination, ultrasound, and/or diagnostic laparoscopy, primary and secondary dysmenorrhea cannot be fully differentiated [4], and the two types share similar symptomatic treatment approaches [5].

Dysmenorrhea negatively affects women’s quality of life [6] and is the leading cause of absence from school and work among women in the reproductive age [7]. It is also associated with other pain conditions such as migraines, fibromyalgia, and irritable bowel syndrome [8–11]. Women with dysmenorrhea, compared to those without, have been reported to have enhanced pain sensitivity, which may increase their susceptibility to develop other chronic pain conditions later in life [6, 12, 13]. Scholars suggest that dysmenorrhea may be a fundamental contributing factor to other painful conditions that are more prevalent in women [9, 14].

Quantitative studies on dysmenorrhea have shown that (1) severe pain is experienced by 2–29% of women with dysmenorrhea [2], (2) gastrointestinal symptoms are prevalent among women with menstrual pain [15, 16] and (3) women use pharmacological and nonpharmacological strategies to manage dysmenorrhea [17, 18]. Relevant qualitative studies have focused primarily on endometriosis [19–21], a source of secondary dysmenorrhea. Women often experience significant delays in the diagnosis of endometriosis from the time of symptom onset [20]. These studies also suggest that endometriosis-related pain and infertility can negatively affect women’s social, work, and sex life [20, 21]. Women in these studies, however, were often recruited from specialized clinics and women with dysmenorrhea symptoms without a clinical diagnosis or who were not in treatment were not included. The goal of the one qualitative study on primary and secondary dysmenorrhea was to develop an outcome measure to support labeling claims in pharmaceutical trials [22].

Research is warranted to address the needs and issues that are important to women with dysmenorrhea to improve their quality of life. The purpose of this study, therefore, was to describe women’s salient thoughts about their experiences of dysmenorrhea. Such information is foundational for the assessment of dysmenorrhea and the development of person-centered interventions to support dysmenorrhea management.

## Methods

### Data collection

The study reported here was part of a larger, cross-sectional survey of women with dysmenorrhea that included both quantitative and qualitative data [23]. The quantitative portion of the study examined self-management behaviors and is described elsewhere [23]. Participants of the larger survey were recruited from a list of survey panel registrants (Qualtrics, Provo, UT) who were willing to be contacted for surveys [24]. Women were eligible if they were over 18 years old, self-identified as having experienced dysmenorrhea symptoms in the last 6 months, were able to read and write in English, and were living in the United States.

Following approval from the University of Wisconsin-Madison Health Sciences Institutional Review Board, the survey provider used demographic data on file to identify potentially eligible participants and sent them e-mail invitations to participate in the study. Women who were interested in participating were informed that completion of the survey implied consent to participate. A screening question followed to determine if they had experienced dysmenorrhea symptoms in the last 6 months. Data were collected in January and February 2015. The survey was completed by 762 women.

The larger survey contained items on demographics, clinical characteristics, and dysmenorrhea self-management behaviors, as well as one open-ended question at the end. The question read “Please write anything else you’d like to share with us about your dysmenorrhea experience.” We made the assumption that responses to the open-ended question reflected what was important to women and represented their salient thoughts about their dysmenorrhea experiences. For the purpose of the study reported here, the responses to the open-ended question were analyzed.

### Data analysis

The data were analyzed by three team members who are nurse scientists with expertise in women’s health and/or qualitative methods. Thematic analysis, as described by Braun and Clarke [25], was used to analyze the responses to the open-ended question. In this analysis, we used a systematic process to find patterned responses or themes within a narrative data set [25]. We followed the 6 stage-analysis provided by Braun and Clarke [25]:

1. Familiarizing yourself with the data*.* All open-ended responses were read repeatedly by the research team members to obtain a sense of the breadth and depth of the data. The team members met on several occasions to discuss their initial impressions of the data.
2. Generating initial codes: All relevant phrases, sentences, or paragraphs (text units) that related to the research question were extracted and given a code, which is a brief label that captures the essence of the text unit. The team discussed, refined, and verified all the codes. The codes were then organized in a data display table.
3. Searching for themes. The team divided the codes into broad overarching themes based on code similarities. Visual representations were developed to explore the relationships among codes within each of the themes.
4. Reviewing themes. The themes were reviewed and revised by the team and organized into a coherent pattern. A coherent pattern includes internal homogeneity (i.e., the codes link together meaningfully in each theme) and external heterogeneity (i.e., there are clear distinctions between the themes). The team then reexamined the narrative data set as a whole to ensure that all relevant data were captured by one of the themes.
5. Defining and naming themes. Each theme was identified by a statement that captured a distinct aspect of the dysmenorrhea experience as described by women.
6. Producing the report. The final report that provided a detailed account of each theme was prepared.

Trustworthiness of findings was established through peer debriefing. Peer debriefing was used at several stages in the process as two team members (CC and JC) conducted the initial analysis and a third team member (CD) independently validated their conclusions with a reexamination of data. In addition, one team member maintained an audit trail to chronicle all methodological and analytic decisions and this audit trail was routinely reviewed by other team members. All data display tables and visual representations of the evolving findings were retained for the audit trail.

## Results

### Sample description

Among 762 survey participants, 311 (40.81%) entered responses to the open-ended question. Eighty-six did not directly address the question (e.g., “This is an interesting research survey. I hope my opinions mattered”), and their responses were removed from the analysis. The final sample consisted of 225 (29.53%) women, and their responses were included for the thematic analysis.

Women in the final sample had a mean age of 34.7 years (SD = 6.8, Range = 18 to 57). Most were White/Caucasian (73.8%), and 93.3% grew up in the United States. About one third had a bachelor’s degree or above (36.4%), and the majority had insurance (86.7%). About one-quarter (27.8%) reported having been diagnosed with conditions related to secondary dysmenorrhea.

As shown in Table 1, age, race, ethnicity, educational level, and medication usage rate were similar between women who provided usable responses and women who did not. However, compared to women who did not provide usable responses, women who did were more likely to (1) have grown up outside of the United States, (2) report more severe dysmenorrhea symptoms, and (3) report being diagnosed with a condition related to secondary dysmenorrhea.

**Table 1:** Demographics comparison between participants with usable responses to the open-ended question and participants without usable responses

	Participants with usable responses (*N* = 225)	Participants without usable responses (*N* = 537)		United States Census Data (%) [36]
	Mean (SD)	Mean (SD)	*p* ^*^	
Age, years	34.8 (6.7)	33.8 (6.4)	0.09	
Dysmenorrhea symptom severity (0 “not severe at all” -10 “extremely severe”)	6.7 (2.2)	6.0 (2.1)	< 0.01	
	n (%)	n (%)	*p* ^**^	
Hispanic Ethnicity	26 (11.6)	52 (9.7)	0.44	17.6
Race				
Asian/Pacific Islander	12 (5.3)	33 (6.2)	0.66	5.6
Black/African American	29 (12.9)	79 (14.7)	0.51	13.3
White/Caucasian	166 (73.8)	410 (76.4)	0.45	77.1
Native American	7 (3.1)	8 (1.5)	0.16	1.2
Bachelor’s degree or above	82 (36.4)	215 (40.0)	0.35	29.3
Had diagnosed with conditions related to secondary dysmenorrhea	49 (27.8)	88 (16.4)	0.08	
Uterine Fibroids	22 (9.8)	37 (6.9)	0.17	
Endometriosis	12 (5.3)	31 (5.8)	0.81	
Pelvic Inflammatory Disease	11 (4.9)	15 (2.5)	0.14	
Others	8 (3.6)	19 (3.6)	0.99	
Insured	195 (86.7)	457 (85.1)	0.65	
Grew up in the United States	210 (93.3)	522 (97.2)	0.01	
Diagnosed with a condition related to secondary dysmenorrhea	50 (22.2)	87 (16.2)	.05	
Used Medications for Dysmenorrhea in the Last 6 Months	172 (76.4)	385 (71.7)	0.18	

*Based on independent samples t-test comparison between participants with and without usable responses

** Based on χ ^2^ tests between participants with and without usable responses

### Themes reflecting women’s salient thoughts on dysmenorrhea

Six themes related to women’s salient thoughts about dysmenorrhea experiences were identified. Each theme is described below.

#### Theme 1: The dysmenorrhea symptom experience varied among the women

The women described their symptoms in a wide variety of ways. Some women specified the location of their pain; most had abdominal pain, but some also experienced pain in the lower back, legs, vagina, and head. A few had “pain all over.” Women commonly had gastrointestinal symptoms including “menstrual nausea,” vomiting, gas, bloating, diarrhea, and “bubbling gut.” These symptoms could occur with or independently of menstrual pain. One participant, for example, wrote, “For me, it’s really the strong increase in bowel movements that I notice.”

The women indicated that the symptom severity ranged from “never bad” to “unbearable,” “horrible,” and “excruciating.” One woman wrote that her menstrual pain was “worse than vaginal delivery.” A few women described their symptoms as “annoying,” “gross,” “haunting,” and “worrisome,” causing some to “dread” their menstrual periods. Several wished for menopause or a hysterectomy to end the pain. A few women had such severe pain they had visited emergency rooms to obtain analgesics.

The women often compared their symptoms to those of their family members and friends. One woman stated, “My friends and I all compare period stories.” Several women stressed that each woman’s experience was unique. One stated, “All I know is that none of us experience exactly the same thing nor [are] our pain levels the same. Treating all of us the same doesn’t work because we aren’t the same.”

#### Theme 2: The dysmenorrhea symptom experience varied across time

The women described a number of ways in which dysmenorrhea symptoms unfolded over time. They indicated that the type and severity of symptoms often changed from day to day during the menstrual cycle. One woman wrote, “I get pain and cramps a week before my period… low back pain, cramps, stomach cramping, diarrhea, etc., the first day, nausea and migraines the second day.”

Others described changes from one menstrual cycle to another. They had symptoms in some months, but not other months, and symptoms could vary in type or severity from cycle to cycle. One woman wrote that her cycles were “never the same, in terms of how much pain comes and what symptoms occur.” Another wrote, “Mine [my pain] is every other period, one month it’s super light and not really painful, then the next month it’s horrible! For years it’s been like this.”

Some women indicated that their symptoms had changed over the years. These women described increasing, decreasing, fluctuating, or stable symptoms in relation to pregnancy, childbearing, aging, and/or menopause. Some had heard from other women that dysmenorrhea symptoms would decrease with aging and childbearing but were disappointed when this did not occur. One wrote, “I keep hoping that as I get older it would get better, but it has gone just the opposite,” and another wrote, “I was told back then in Africa that the dysmenorrhea would stop completely once I have kids. An old wives’ tale I guess, because this is definitely not true in my case.”

#### Theme 3: A variety of factors influenced the dysmenorrhea symptom experience

The women identified several factors that contributed to or exacerbated dysmenorrhea symptoms. They indicated that certain characteristics of the menstrual cycle, including “heavy periods” and menstrual irregularity, worsened dysmenorrhea symptoms. One woman wrote, “My menstrual cycle has never been regular. The longer I go without having a period (e.g., 2 months or more), the more intense my symptoms become, and my flow itself picks up a lot as well.” In some cases, the menstrual irregularities that aggravated their symptoms occurred during perimenopause.

Some attributed symptoms to other diagnosed health conditions such as endometriosis, sex hormone imbalances, or uterine fibroids. Others believed a suspected but an undiagnosed health condition worsened their symptoms. One woman wrote, “I believe I have endometriosis, although no one has ever diagnosed me with it. My symptoms match with it.” Other women indicated that their dysmenorrhea symptoms were caused or exacerbated by genetics or heredity, dietary habits (e.g., “eating cold food or drinking cold drink”), and the recent removal of intrauterine devices.

#### Theme 4: Dysmenorrhea symptoms could have a negative impact on the women’s daily lives

The women indicated that dysmenorrhea had adversely affected their daily lives in a number of ways. Some were unable to sit, walk, or stand when they had dysmenorrhea symptoms. Others could not leave home and would lie in bed or “curl up in a ball.” Some could not attend school or work, enjoy recreational activities (“skipping parties”), and attend to family responsibilities (e.g. “dealing with kids”) because of their severe symptoms.

A few women indicated that the severity of their sympoms put them at risk for medication overdose. One woman wrote, “This was not intentional but the cramps were so bad I just kept taking more meds….” Some women suggested that they were desperate to get relief from their pain. One woman wrote, “I used to have burn marks on my abdomen because the heating pads I used were too hot, but that was the only thing that helped.”

#### Theme 5: Dysmenorrhea was not always seen as a legitimate health issue

Some women did not view dysmenorrhea as a legitimate health issue. They indicated that they thought of their symptoms as “part of life,” “part of a menstrual cycle,” or “going along with being a woman.” Several expressed surprise that the symptoms they had been experiencing “actually had a name,” and some were unaware there were treatments available that might provide relief.

The women also revealed that healthcare providers, employers, and society did not view dysmenorrhea as a legitimate problem and often showed little sympathy for women with dysmenorrhea. Several women had discussed their symptoms with doctors who did not deem the symptoms to be severe enough to warrant treatment. One woman suggested to her doctor that she might have endometriosis, but he just “brushed it off.” Another woman wrote that “cramps are hard to deal with, but male bosses (and even some female ones) aren’t very sympathetic.” Some women suggested that society in general had little understanding of the toll dysmenorrhea symptoms can have on women. One woman wrote:

*I think that more women experience these symptoms than [they] will admit, because we don’t want to be seen as the weaker sex. The truth is that menstruation (as well as childbearing) have a significant effect on our bodies…. but there is not enough empathy or understanding for the physical price we pay.*

#### Theme 6: Treatments for the women with dysmenorrhea varied in acceptability and effectiveness

The women had a variety of views on and experiences with dysmenorrhea treatment. Some with severe symptoms for whom pregnancy was not a concern tried hormonal contraceptives and found they not only relieved their pain but also regulated their menstrual cycles. Others were not willing to use “chemical” means to treat dysmenorrhea, found hormonal contraceptives to be ineffective, experienced unfavorable side effects (e.g., diarrhea, weight gain, mood swings, prolonged periods, osteoporosis from long-term Depo-Provera use), or could not use hormonal contraceptives because of age-related risks. Some women found over-the-counter pain medications to be effective, whereas others complained these medications “just take the edge off.” A few experienced relief from endometrial ablation, which was described by one woman as a short “god-sent” procedure with fast recovery time. Others favored “natural treatments” including exercise, healthy eating, eating in smaller portions, and adequate water intake to help with dysmenorrhea symptoms. Some identified the need for more research to develop treatment options that are effective, affordable, and accessible to all women.

## Discussion

This study revealed salient thoughts women have about their dysmenorrhea experiences in the form of six descriptive themes. Limited research has examined women’s experience with dysmenorrhea using qualitative data. By systematically analyzing qualitative data, we newly identified needs and issues that are important from women’s own perspectives. Women’s own perspectives are essential to inform person-centered care for dysmenorrhea. Our study provided new information about the complex, dynamic, and heterogeneous nature of dysmenorrhea. By describing the heterogeneity of the somatology, topography of women’s symptom patterns, the ways in which the symptoms affect their daily lives, their beliefs about contributing factors, and their perceptions about treatment, these findings provide an in-depth understanding of dysmenorrhea as experienced by a broad-based sample of women.

The findings serve as a call for action to improve dysmenorrhea assessment. Abdominal pain intensity is typically the only symptom assessed in dysmenorrhea [26]. Yet consistent with previous studies [16, 22], we found that many participants experienced pain at multiple sites and reported a variety of gastrointestinal symptoms. Previous studies have reported that increased prostaglandins and pain sensitization among women with dysmenorrhea are likely to contribute to pain at multiple sites and gastrointestinal symptoms [3, 11, 27]. Assessment for dysmenorrhea should therefore include the evaluation of pain at different locations (e.g., abdomen, lower back, legs/upper thighs, vaginal area, and head) and query about gastrointestinal symptoms (e.g., nausea, vomiting, diarrhea, and bloating).

Interference of symptoms with daily life has been recognized as a core outcome of pain research and clinical care [28], but it has not been commonly assessed in dysmenorrhea studies [29]. Our study along with others focusing on endometriosis [19, 22] demonstrate that dysmenorrhea symptoms interfere with daily life in a variety of domains (e.g., physical, occupational, recreational, and relational). Such domains are included in interference scales in available instruments such as the Brief Pain Inventory [30] and the Patient-Reported Outcomes Measurement Information System (PROMIS®) [31]. Future studies should evaluate the applicability and psychometric properties of these scales on women with dysmenorrhea.

Although cross-sectional epidemiological studies have uncovered population-based correlates of dysmenorrhea such as age and parity [2, 32, 33], our findings suggest that dysmenorrhea symptoms may fluctuate over time with some women reporting increased rather than decreased symptoms with childbearing, aging, and menopause. The sub-pattern of increased dysmenorrhea symptoms with childbearing, aging, and menopause has rarely been reported in the literature. Longitudinal studies that could explicate sub-patterns of symptom trajectories and allow researchers to identify protective and risk factors for these trajectories and/or the development of chronic pain later in life are needed.

Our findings call for the development of personalized interventions that target the full range of dysmenorrhea symptoms. Clinical guidelines [34] that address the management of menstrual pain and related gastrointestinal symptoms, and effective pharmacological and nonpharmacological treatments for dysmenorrhea-related gastrointestinal symptoms are needed. Given the complex nature of dysmenorrhea, multimodal approaches that combine pharmacological and complementary approaches may be additively or synergistically beneficial for some women. A personalized decision aid could help women choose from among the array of available treatments. The well-established Ottawa Decision Support Framework [35] indicates that treatment decisions should cater for individuals’ needs (e.g., needs for pregnancy/contraception), preferences (e.g., desire for natural treatment and desire for menstruation), expectations, goals, previous treatment responses, and treatment risks. Decision aids could improve dysmenorrhea treatment by validating women’s concerns, clarifying their needs and preferences, and prompting shared decision-making with the healthcare provider.

More mechanistic research is needed to understand individual variations in symptom experiences, symptom trajectories, and treatment responsiveness. Despite advances in explicating the role of prostaglandins in the etiology of dysmenorrhea, it is unclear why some women with severe dysmenorrhea symptoms have normal prostaglandin levels and laparoscopic findings [3]. Uncovering mechanisms underlying this heterogeneity can generate new insights for developing mechanism-specific therapies and tailoring therapies for distinct groups.

The findings of this study have implications for clinical practice. First, healthcare providers should not trivialize dysmenorrhea. Although not life threatening, dysmenorrhea can significantly interfere with the daily lives of some women. Healthcare providers’ indifference toward dysmenorrhea can lead to frustration, delayed diagnosis, and inadequate or ineffective treatment [19]. A vital step toward effective dysmenorrhea management is to validate women’s experience. For example, when a woman reports dysmenorrhea symptoms, a provider might respond by saying “Menstrual pain is very common, but it can be distressing and might interfere with your well-being. I am here to do my best to relieve your symptoms.” Second, differences between women should be given full consideration. Clinical assessment should include questions about dysmenorrhea severity and progression, concurrent problems (e.g., heavy menstruation), treatment preferences (including desire for menstruation, needs for pregnancy/ contraception, concerns about medications, and attitudes toward non-medications), treatment contraindications, and history of treatment responsiveness thus allowing treatment tailoring. For example, in women with severe dysmenorrhea concurrent with heavy menstruation, the levonorgestrel-releasing intrauterine device or continuous use of oral contraceptives may suit their needs [34] especially when they are open to the absence of menstruation. For women who do not desire contraception or who prefer non-pharmaceutical approaches, high-intensity transcutaneous electrical nerve stimulation and/or heat may be reasonable options [5, 34].

These findings should be considered in light of some study limitations. First, given that the responses were anonymous and written in response to an open-ended survey question, we were unable to ask clarifying questions or to validate our conclusions with participants. Second, there was potential recall bias in women’s description of their symptoms. Longitudinal research using standardized symptom measures could explicate symptom patterns across time. Third, there was potential coverage and self-selection bias related to using the Internet survey. Fourth, our broad-based community sample served both as strength and limitation. While the sample allowed us to describe a range of experiences, it did not allow us to make claims about any specific sub-groups of women with dysmenorrhea, such as those who have endometriosis or fibroids. A purposive sample that systematically includes subgroups of women would allow such a comparison.

## Conclusions

In conclusion, this study provides new information on the complex, dynamic, and heterogeneous nature of dysmenorrhea. The findings reported here have implications for dysmenorrhea symptom assessment, development of personalized interventions, and future mechanistic studies. This study underscores the importance of dysmenorrhea in women’s health and highlights the need for additional research and attention to this problem in clinical practice.

## Abbreviation

PROMIS: The Patient-Reported Outcomes Measurement Information System
